# Supplementary material for: Single-cell meta-analysis of T cells reveals clonal dynamics of response to checkpoint immunotherapy
Source: Cell Genom. 2025 Apr 4;5(5):100842. doi: 10.1016/j.xgen.2025.100842 (PMC12143341; doi:10.1016/j.xgen.2025.100842)
Supplement: Document S1. Figures S1–S21 [file mmc1.pdf]

Cell Genomics, Volume *5*

Supplemental information

Single-cell meta-analysis of T cells reveals clonal dynamics of response to checkpoint immunotherapy

Ofir Shorer, Asaf Pinhasi, and Keren Yizhak

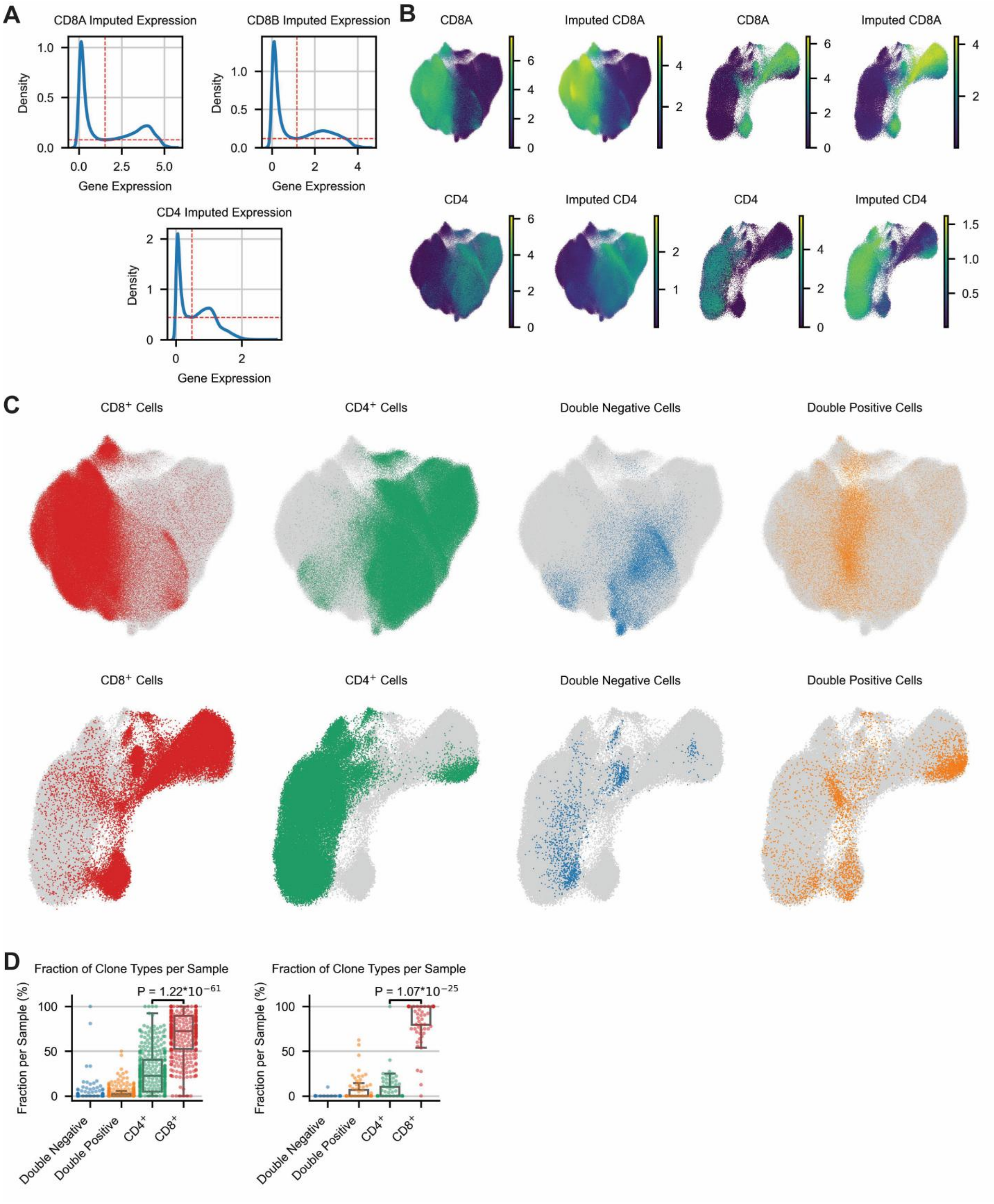

Figure S1. Markov Affinity-based Graph Imputation of Cells (MAGIC [S1]) for detection of drop-outs, Related to Figure 1. A. Bimodal density curves for MAGIC-imputed gene expression of *CD8A/B* and *CD4*. Vertical red dashed lines represent expression threshold. B. UMAP plots showing imputed and non-imputed gene expression of *CD8A* and *CD4* for single cells from tumor samples (left) and blood samples (right). C. Labeling of single cells by four subtypes according to imputed and non-imputed gene expression in tumor

samples (top) and blood samples (bottom). D. Fraction of clone subtypes out of all the expanded clones per sample for tumor samples (left) and blood samples (right).

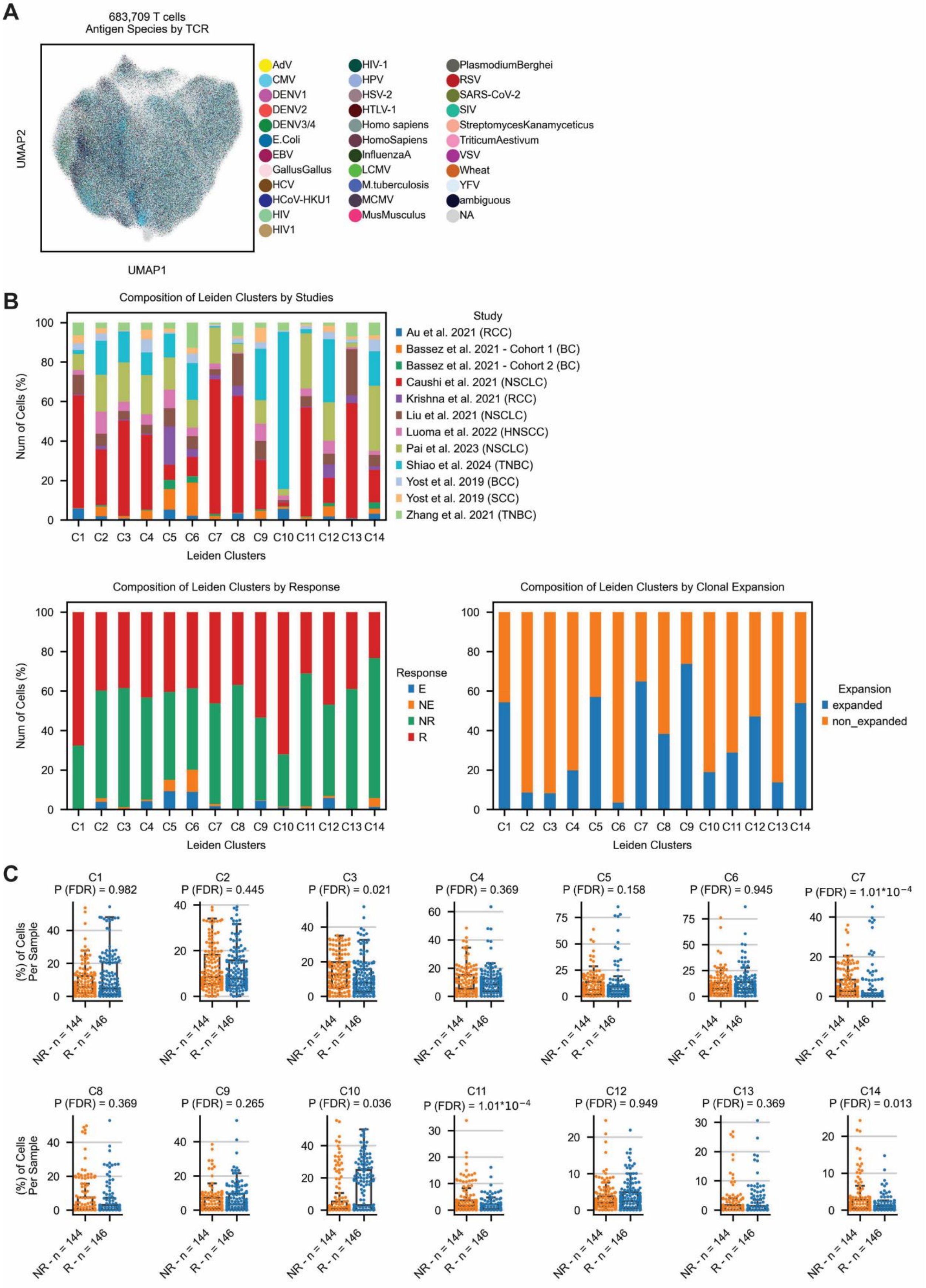
A
683,709 T cells
Antigen Species by TCR
UMAP2
UMAP1
AdV
CMV
DENV1
DENV2
DENV3/4
E.Coli
EBV
GallusGallus
HCV
HCoV-HKU1
HIV
HIV1
HIV-1
HPV
HSV-2
HTLV-1
Homo sapiens
HomoSapiens
InfluenzaA
LCMV
M.tuberculosis
MCMV
MusMusculus
PlasmodiumBerghei
RSV
SARS-CoV-2
SIV
StreptomycesKanamyceticus
TriticumAestivum
VSV
Wheat
YFV
ambiguous
NA
B
Composition of Leiden Clusters by Studies
Num of Cells (%)
Leiden Clusters
Study
Au et al. 2021 (RCC)
Bassez et al. 2021 - Cohort 1 (BC)
Bassez et al. 2021 - Cohort 2 (BC)
Caushi et al. 2021 (NSCLC)
Krishna et al. 2021 (RCC)
Liu et al. 2021 (NSCLC)
Luoma et al. 2022 (HNSCC)
Pai et al. 2023 (NSCLC)
Shiao et al. 2024 (TNBC)
Yost et al. 2019 (BCC)
Yost et al. 2019 (SCC)
Zhang et al. 2021 (TNBC)
Composition of Leiden Clusters by Response
Response
E
NE
NR
R
Composition of Leiden Clusters by Clonal Expansion
Expansion
expanded
non_expanded
C1 C2 C3 C4 C5 C6 C7 C8 C9 C10 C11 C12 C13 C14
C
C1 P (FDR) = 0.982
C2 P (FDR) = 0.445
C3 P (FDR) = 0.021
C4 P (FDR) = 0.369
C5 P (FDR) = 0.158
C6 P (FDR) = 0.945
C7 P (FDR) = 1.01*10^-4
C8 P (FDR) = 0.369
C9 P (FDR) = 0.265
C10 P (FDR) = 0.036
C11 P (FDR) = 1.01*10^-4
C12 P (FDR) = 0.949
C13 P (FDR) = 0.369
C14 P (FDR) = 0.013
(%) of Cells Per Sample
NR - n = 144
R - n = 146

Figure S2. Paired scRNA/TCRseq of 683,709 T cells from tumor samples of ICI-treated patients, Related to Figure 1. A. UMAP plot of antigen species attributed to each single cell following epitope annotations using VDJdb [S2]. B. Composition of 14 Leiden clusters by single-cell study, clinical outcome, and clonal expansion. C. Percentage of T cells found in clusters associated with patient response, separated by their response status. Note that samples from Bassez et al. [S3] do not have annotations for clinical outcome and were therefore not considered for this analysis. Abbreviations: R = Responders, NR = Non-responders, E/NE = Patient-level annotations for clonal expansion by Bassez et al. [S3].

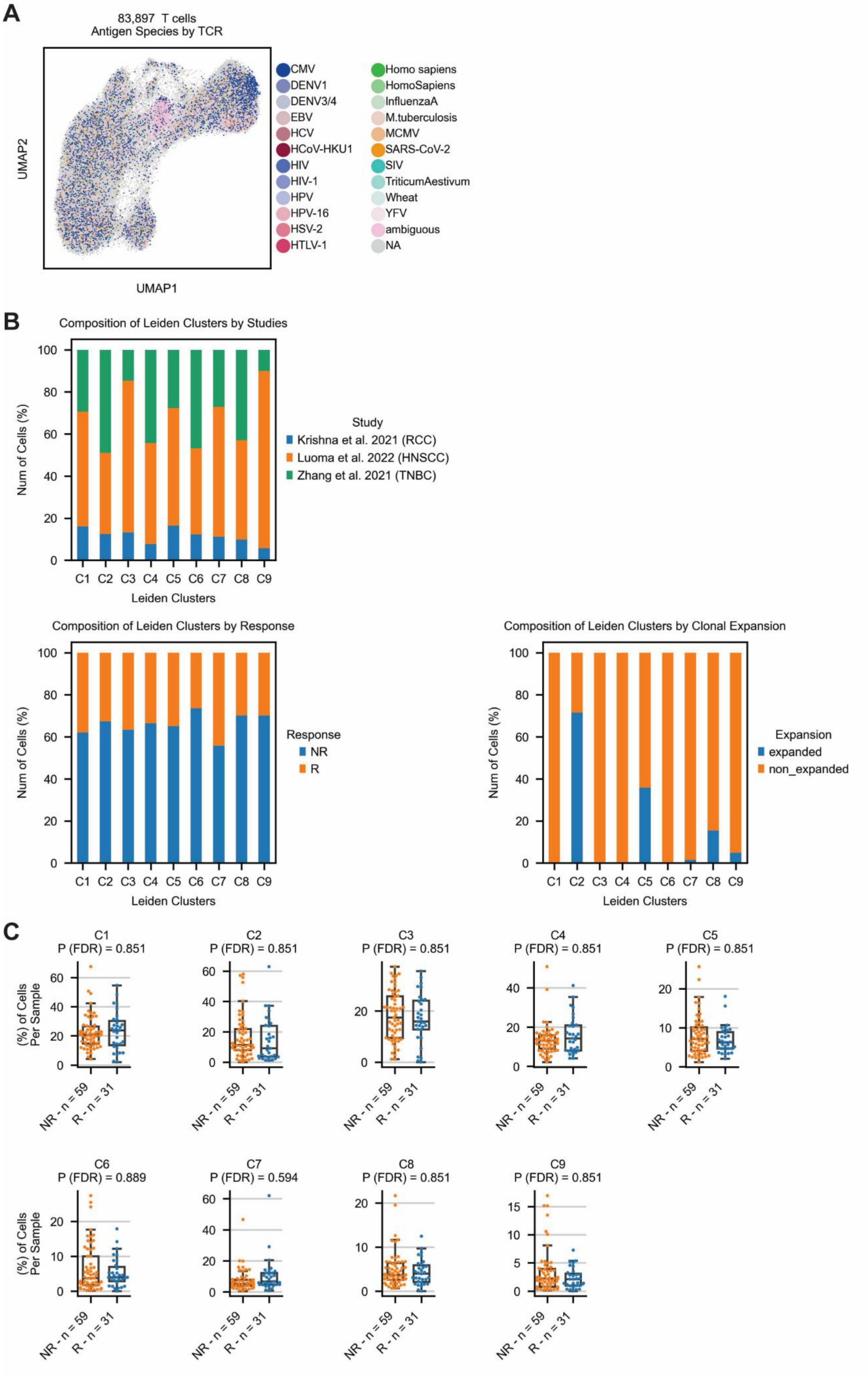
A
83,897 T cells
Antigen Species by TCR
UMAP2
UMAP1
CMV
DENV1
DENV3/4
EBV
HCV
HCoV-HKU1
HIV
HIV-1
HPV
HPV-16
HSV-2
HTLV-1
Homo sapiens
HomoSapiens
InfluenzaA
M.tuberculosis
MCMV
SARS-CoV-2
SIV
TriticumAestivum
Wheat
YFV
ambiguous
NA
B
Composition of Leiden Clusters by Studies
Num of Cells (%)
Leiden Clusters
C1
C2
C3
C4
C5
C6
C7
C8
C9
Study
Krishna et al. 2021 (RCC)
Luoma et al. 2022 (HNSCC)
Zhang et al. 2021 (TNBC)
Composition of Leiden Clusters by Response
Response
NR
R
Composition of Leiden Clusters by Clonal Expansion
Expansion
expanded
non_expanded
C
C1
P (FDR) = 0.851
C2
P (FDR) = 0.851
C3
P (FDR) = 0.851
C4
P (FDR) = 0.851
C5
P (FDR) = 0.851
C6
P (FDR) = 0.889
C7
P (FDR) = 0.594
C8
P (FDR) = 0.851
C9
P (FDR) = 0.851
(%) of Cells Per Sample
NR - n = 59
R - n = 31

Figure S3. Paired scRNA/TCRseq of 83,897 T cells from blood samples of ICI-treated patients, Related to Figure 1. A. UMAP plot of antigen species attributed to each single cell following epitope annotations using VDJdb [S2]. B. Composition of 9 Leiden clusters by single-cell study, clinical outcome, and clonal expansion. C. Percentage of T cells found in clusters associated with patient response, separated by their response status. Abbreviations: R = Responders, NR = Non-responders.

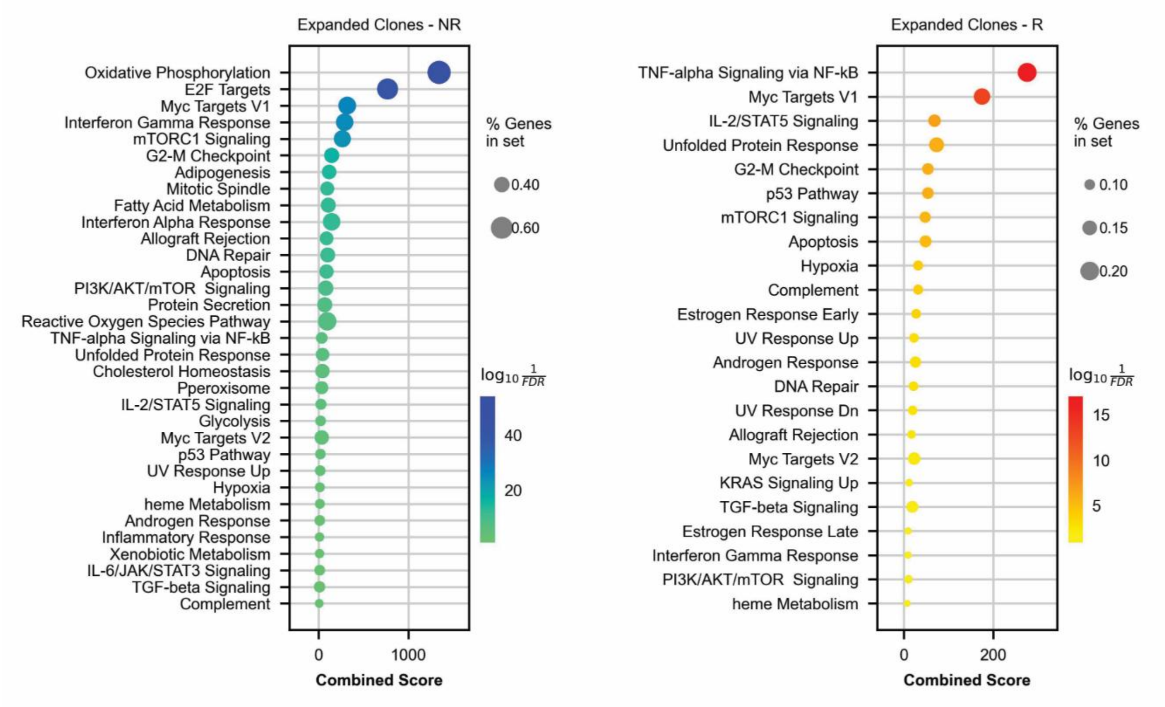

Figure S4. Pathway enrichment analysis of expanded CD8⁺ T cells in tumor samples, between responders (right) and non-responders (left), Related to Figure 1. Abbreviations: R = Responders, NR = Non-responders.

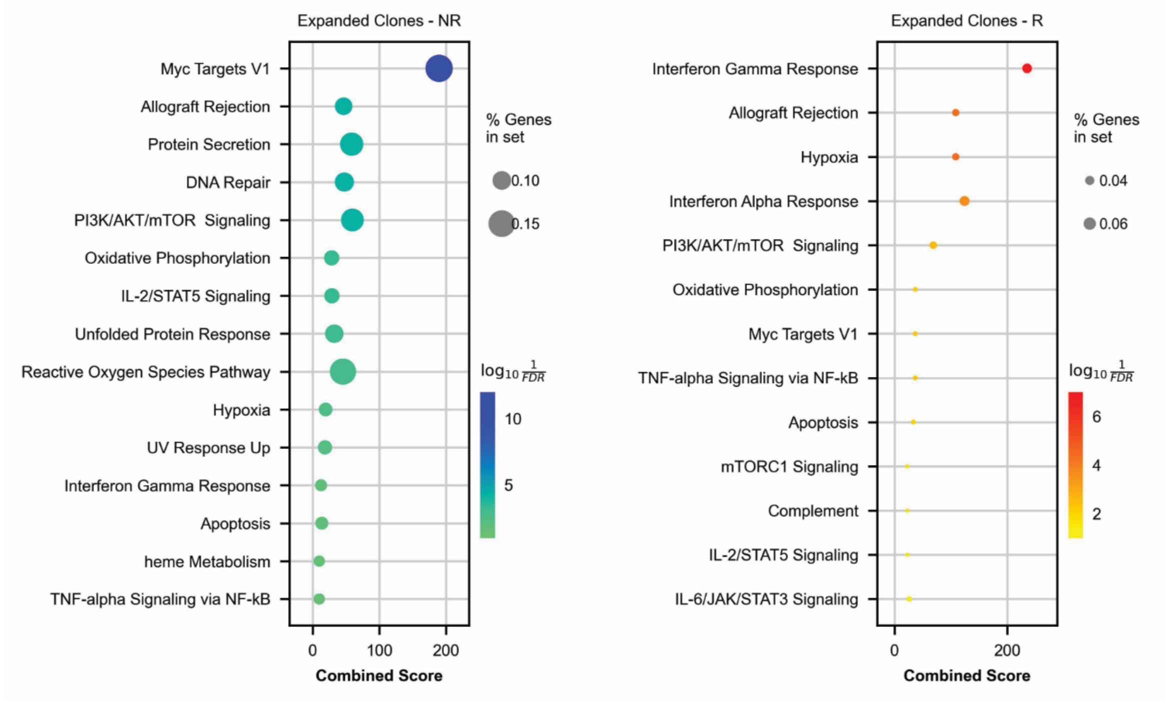

Figure S5. Pathway enrichment analysis of expanded CD8⁺ T cells in blood samples, between responders (right) and non-responders (left), Related to Figure 1. Abbreviations: R = Responders, NR = Non-responders.

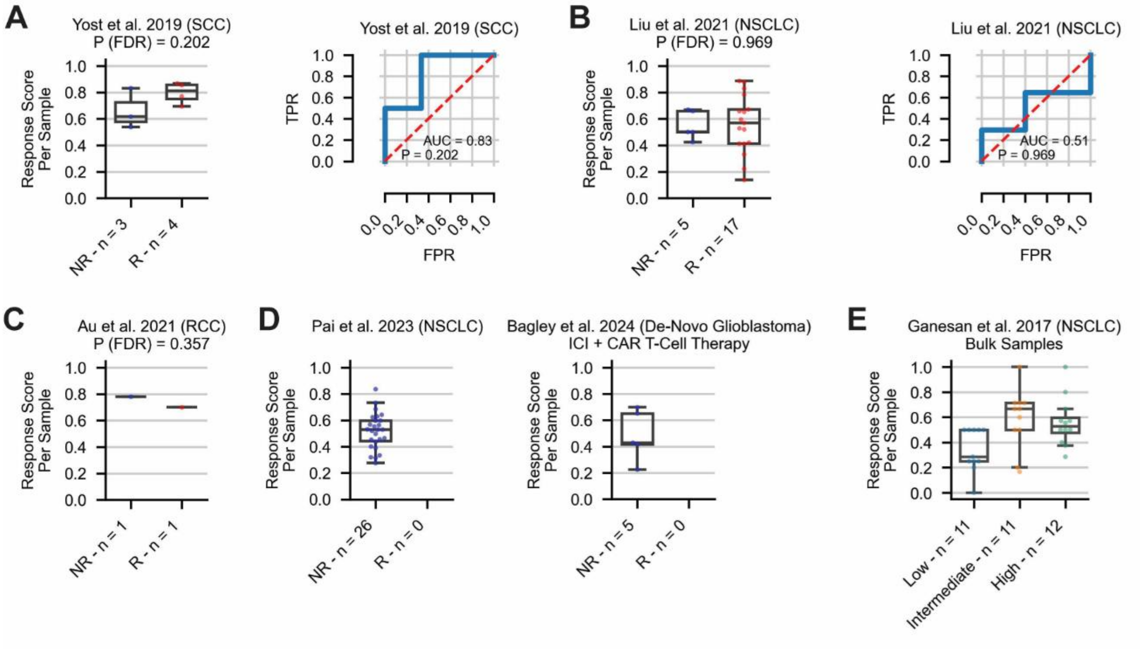

Figure S6. Independent test of the response score per dataset, Related to Figure 2. A. The performance of the response score across expanded CD8+ T cells from tumor samples of SCC patients [S4]. ROC and the corresponding AUC achieved by the response score is shown on the right; Distribution of the response score in responders and non-responders is shown on the left. B. The performance of the response score across expanded CD8+ T cells from tumor samples of NSCLC patients [S5]. ROC and the corresponding AUC achieved by the response score is shown on the right; Distribution of the response score in responders and non-responders is shown on the left. C. The distribution of the response score in responders and non-responders across tumor samples of RCC patients [S6]. D. The distribution of the response score across tumor samples of de-novo glioblastoma patients [S7] (right), and NSCLC patients [S8] (left), all failed to respond. E. The distribution of the response score across sorted bulk samples of NSCLC patients labeled according to the infiltration of CD8+ T cells into the tumor as originally provided by the authors [S9]. Abbreviations: R = Responders, NR = Non-responders, FPR = False positive rate, TPR = True positive rate.

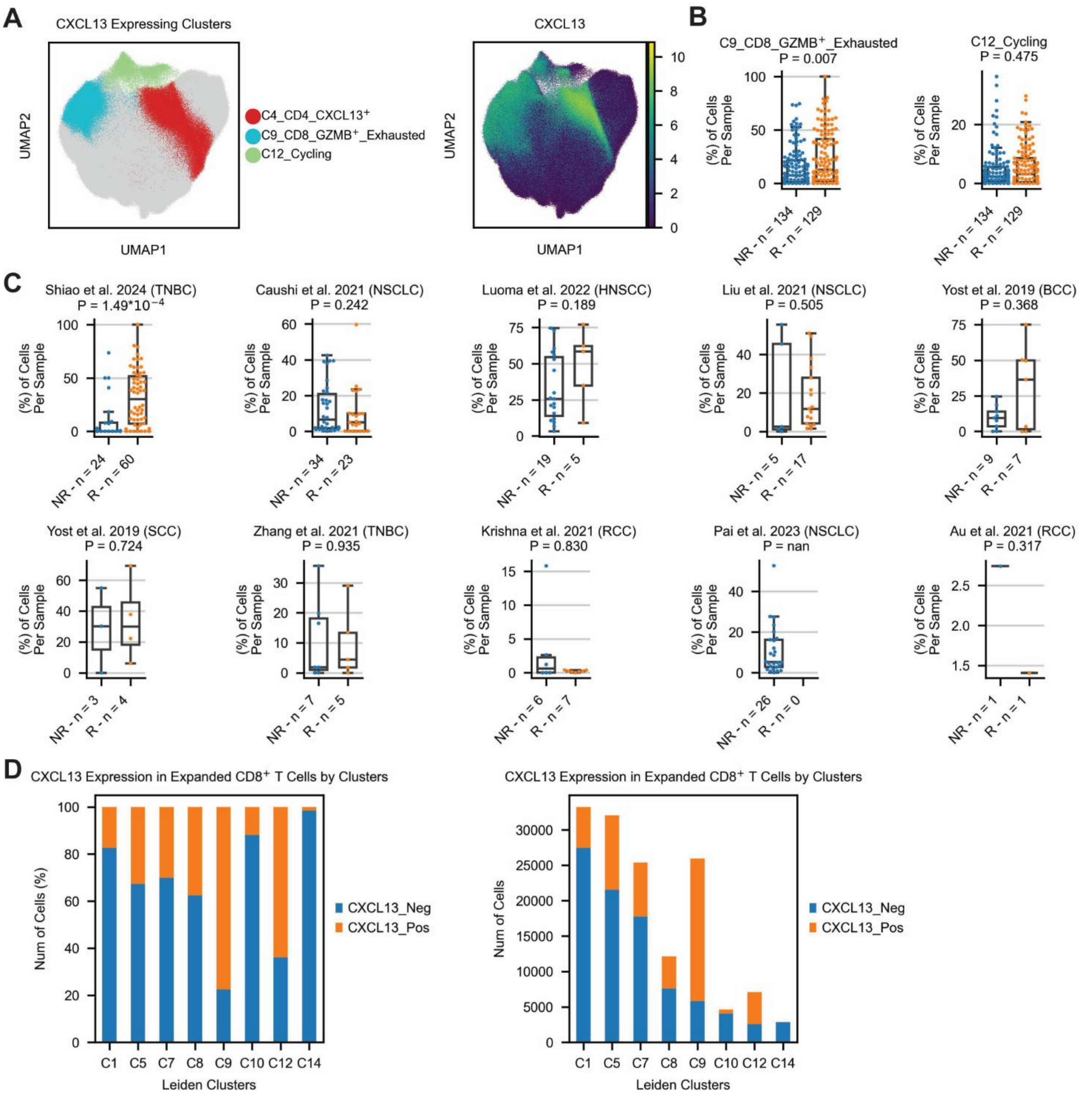

Figure S7. Association of *CXCL13*-expressing CD8^{+} clusters with therapy response, Related to Figure 2. A. UMAP plots depicting *CXCL13*-expressing clusters (left) and *CXCL13* expression across single-cells (right). B. Association of *CXCL13*-expressing CD8^{+} clusters (C9 & C12) with clinical outcome. C. Abundance of single cells from C9 in samples obtained from responders and non-responders, across individual single-cell datasets. D. Amount of CXCL13^{+} and CXCL13^{-} CD8^{+} T cells from expanded clones by Leiden clusters. Results are shown by percentages (left) and by absolute numbers (right). Abbreviations: R = Responders, NR = Non-responders.

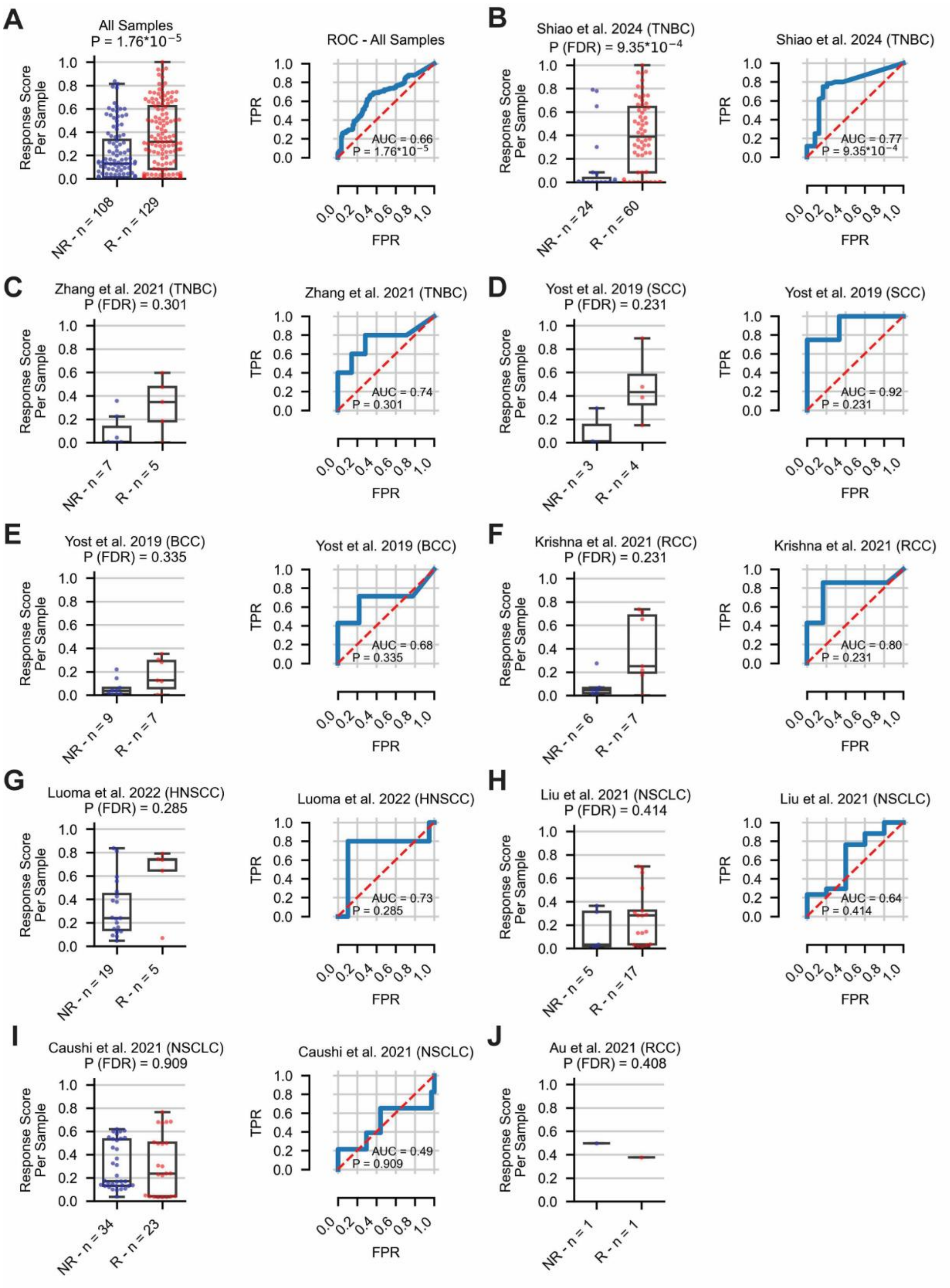

Figure S8. Predictive performance of *CXCL13*-expressing CD8$^+$ T cells from expanded clones across studies, Related to Figure 2. A. The performance of the response score using *CXCL13* expression alone across expanded CD8$^+$ T cells from 237 tumor samples spanning 9 single-cell studies. ROC and the corresponding AUC achieved by the response score is shown on the right; Distribution of the response score in responders and non-responders is shown on the left. B-J. The performance of the response score across expanded CD8$^+$ T cells from tumor samples for individual datasets independently. Abbreviations: R = Responders, NR = Non-responders, FPR = False positive rate, TPR = True positive rate.

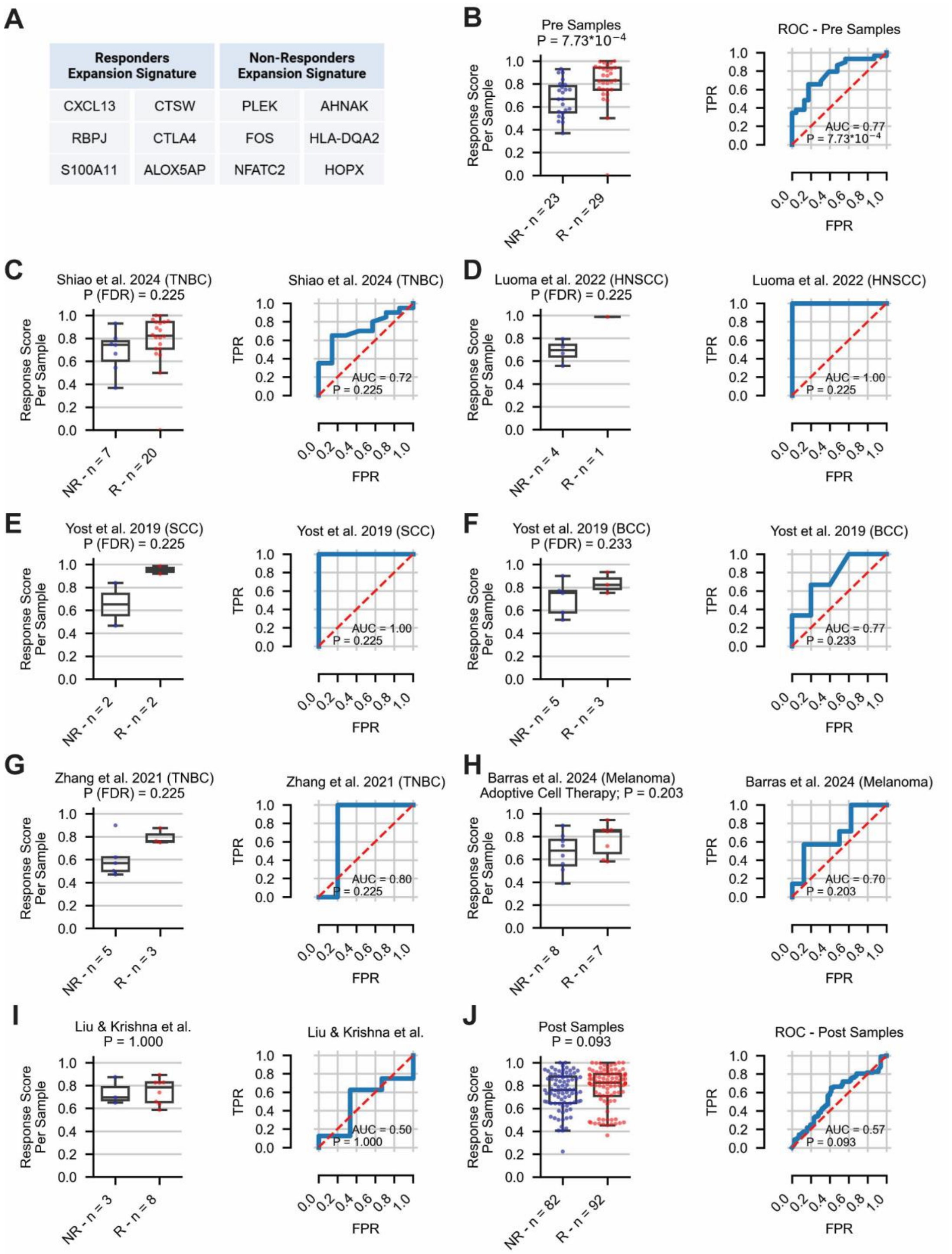

Figure S9. Predictive performance of a response signature constructed using baseline samples alone, Related to Figure 2. A. Robust response signature of 6 markers obtained for responders (left) and non-responders (right), developed using baseline samples. B. The performance of the response score across expanded CD8$^+$ T cells from 52 baseline tumor samples spanning 5 single-cell studies [S4], [S10], [S11], [S12]. ROC and the corresponding AUC achieved by the response score is shown on the right; Distribution of the response score in responders and non-responders is shown on the left. C-G. The performance of the response score across expanded CD8$^+$ T cells from baseline tumor samples of individual discovery datasets independently. H-I. The performance of the response score across expanded

$CD8^+$ T cells from baseline tumor samples of additional validation cohorts [S5], [S13], [S14]. J. The performance of the response score across expanded $CD8^+$ T cells from post-treatment tumor samples. Abbreviations: R = Responders, NR = Non-responders, FPR = False positive rate, TPR = True positive rate.

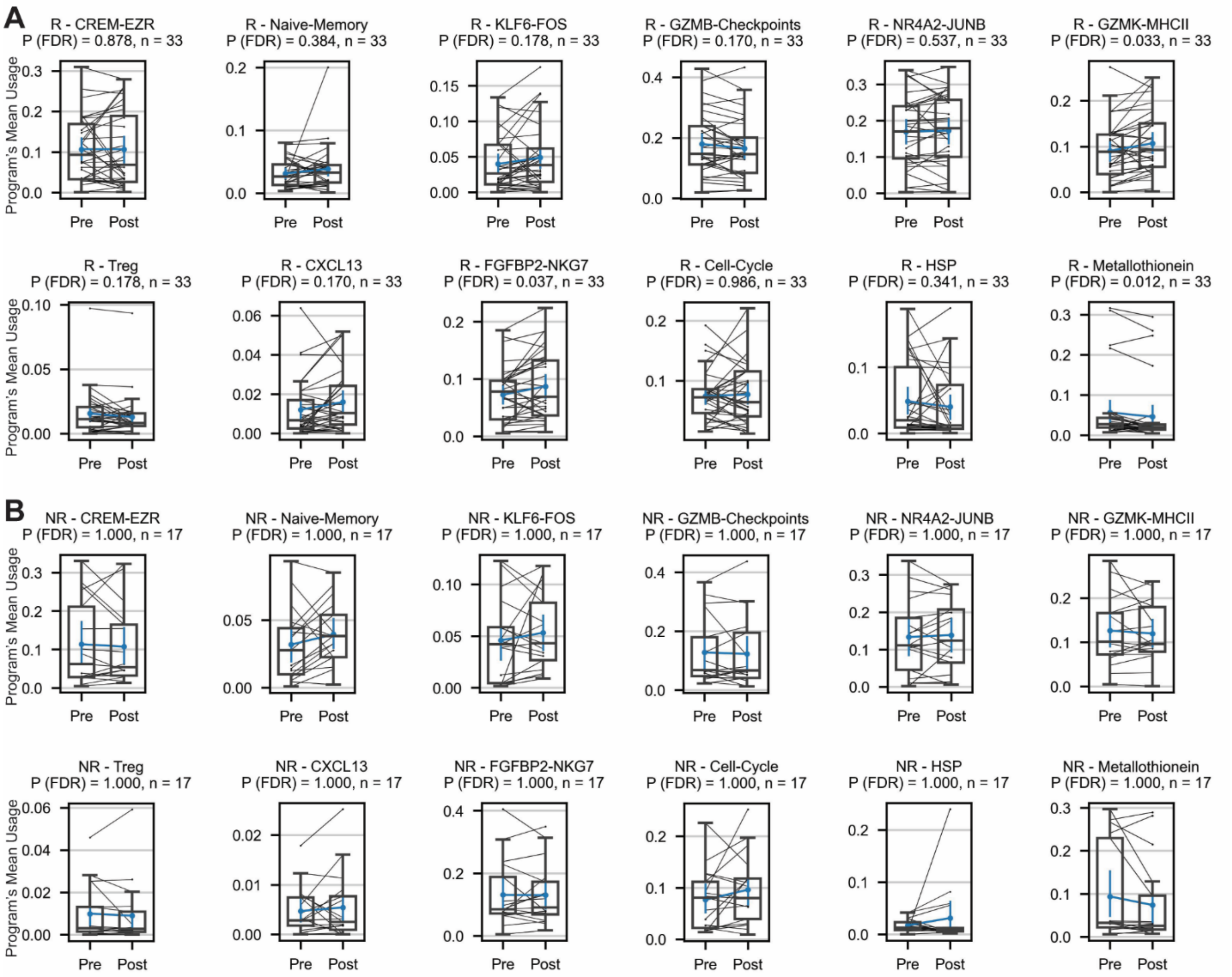

Figure S10. Changes following therapy of cNMF [S15] programs for the top 5 expanded persistent CD8⁺ clones per patient in tumor samples, Related to Figure 3. A. Changes in program activity following therapy for responders. B. Changes in program activity following therapy for non-responders. Abbreviations: R = Responders, NR = Non-responders.

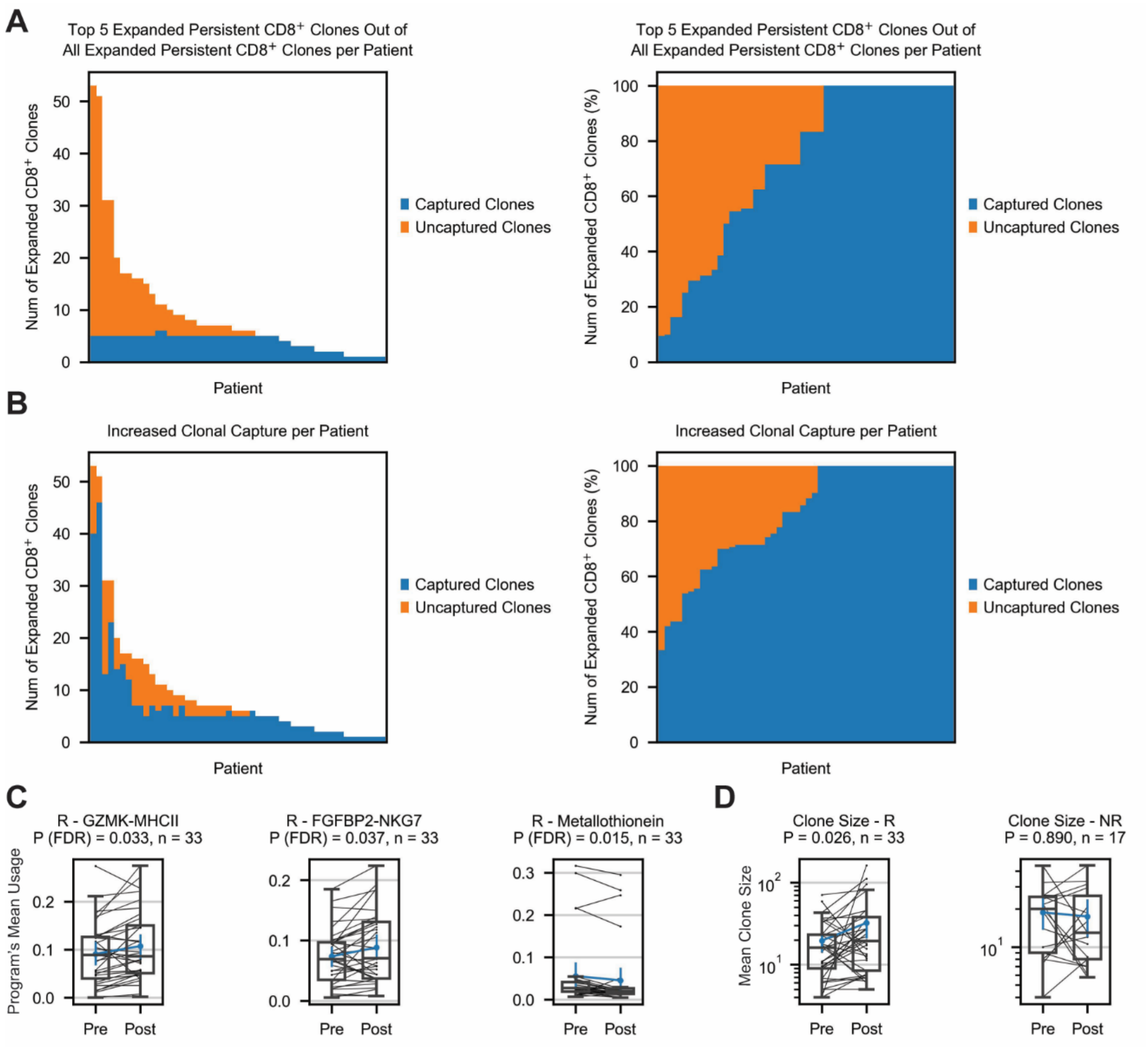

Figure S11. Transcriptional changes within persistent CD8+ clones and their association with clinical outcome following increased clonal capture per patient, Related to Figure 3. A. Original clonal capture of persistent CD8+ clones per patient, including the absolute number of clones that were considered (left), and the normalized amount per patient (right). B. Increased clonal capture per patient, including the absolute number of clones that were considered (left), and the normalized amount per patient (right). C. Changes following therapy of selected transcriptional programs for the increased capture of persistent clones per patient in responders (n = 33 patients). D. Change following therapy of the mean clone size for the increased capture of persistent clones per patient in responders (left, n = 33 patients) and non-responders (right, n = 17 patients). Abbreviations: R = Responders, NR = Non-responders.

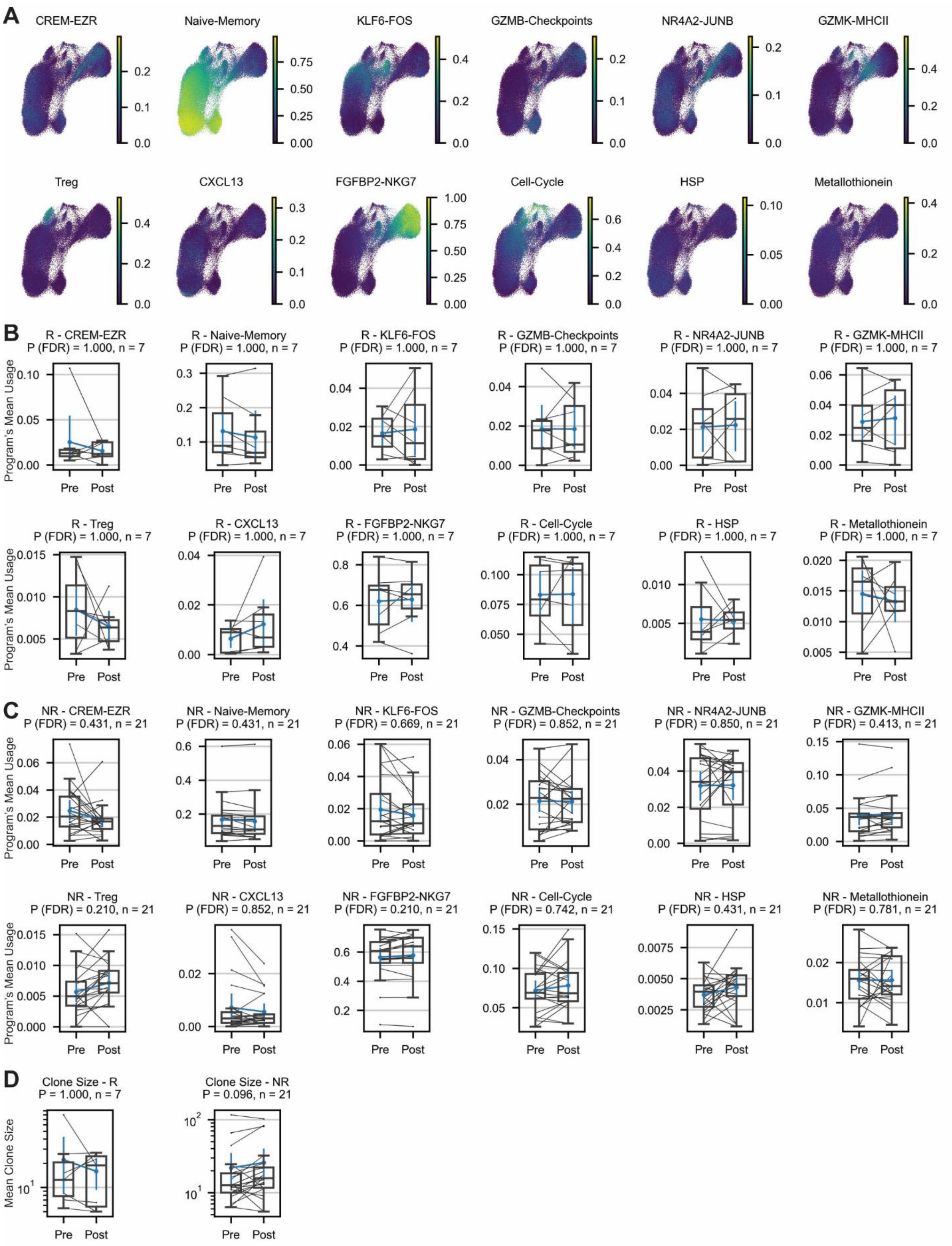

A
CREM-EZR
Naive-Memory
KLF6-FOS
GZMB-Checkpoints
NR4A2-JUNB
GZMK-MHCII
Treg
CXCL13
FGFBP2-NKG7
Cell-Cycle
HSP
Metallothionein
B
R - CREM-EZR
P (FDR) = 1.000, n = 7
R - Naive-Memory
P (FDR) = 1.000, n = 7
R - KLF6-FOS
P (FDR) = 1.000, n = 7
R - GZMB-Checkpoints
P (FDR) = 1.000, n = 7
R - NR4A2-JUNB
P (FDR) = 1.000, n = 7
R - GZMK-MHCII
P (FDR) = 1.000, n = 7
R - Treg
P (FDR) = 1.000, n = 7
R - CXCL13
P (FDR) = 1.000, n = 7
R - FGFBP2-NKG7
P (FDR) = 1.000, n = 7
R - Cell-Cycle
P (FDR) = 1.000, n = 7
R - HSP
P (FDR) = 1.000, n = 7
R - Metallothionein
P (FDR) = 1.000, n = 7
Program's Mean Usage
Pre
Post
C
NR - CREM-EZR
P (FDR) = 0.431, n = 21
NR - Naive-Memory
P (FDR) = 0.431, n = 21
NR - KLF6-FOS
P (FDR) = 0.669, n = 21
NR - GZMB-Checkpoints
P (FDR) = 0.852, n = 21
NR - NR4A2-JUNB
P (FDR) = 0.850, n = 21
NR - GZMK-MHCII
P (FDR) = 0.413, n = 21
NR - Treg
P (FDR) = 0.210, n = 21
NR - CXCL13
P (FDR) = 0.852, n = 21
NR - FGFBP2-NKG7
P (FDR) = 0.210, n = 21
NR - Cell-Cycle
P (FDR) = 0.742, n = 21
NR - HSP
P (FDR) = 0.431, n = 21
NR - Metallothionein
P (FDR) = 0.781, n = 21
D
Clone Size - R
P = 1.000, n = 7
Clone Size - NR
P = 0.096, n = 21
Mean Clone Size

Figure S12. Changes following therapy of cNMF [S15] programs for the top 5 expanded persistent CD8$^+$ clones per patient in blood samples, Related to Figure 3. A. Twelve transcriptional programs obtained using cNMF and their activity across single cells from blood samples. B. Changes in program activity following therapy for responders. C. Changes in program activity following therapy for non-responders. D. Change following therapy of the mean clone size for the top 5 expanded persistent CD8$^+$ clones per patient: responders (left) and non-responders (right). Abbreviations: R = Responders, NR = Non-responders.

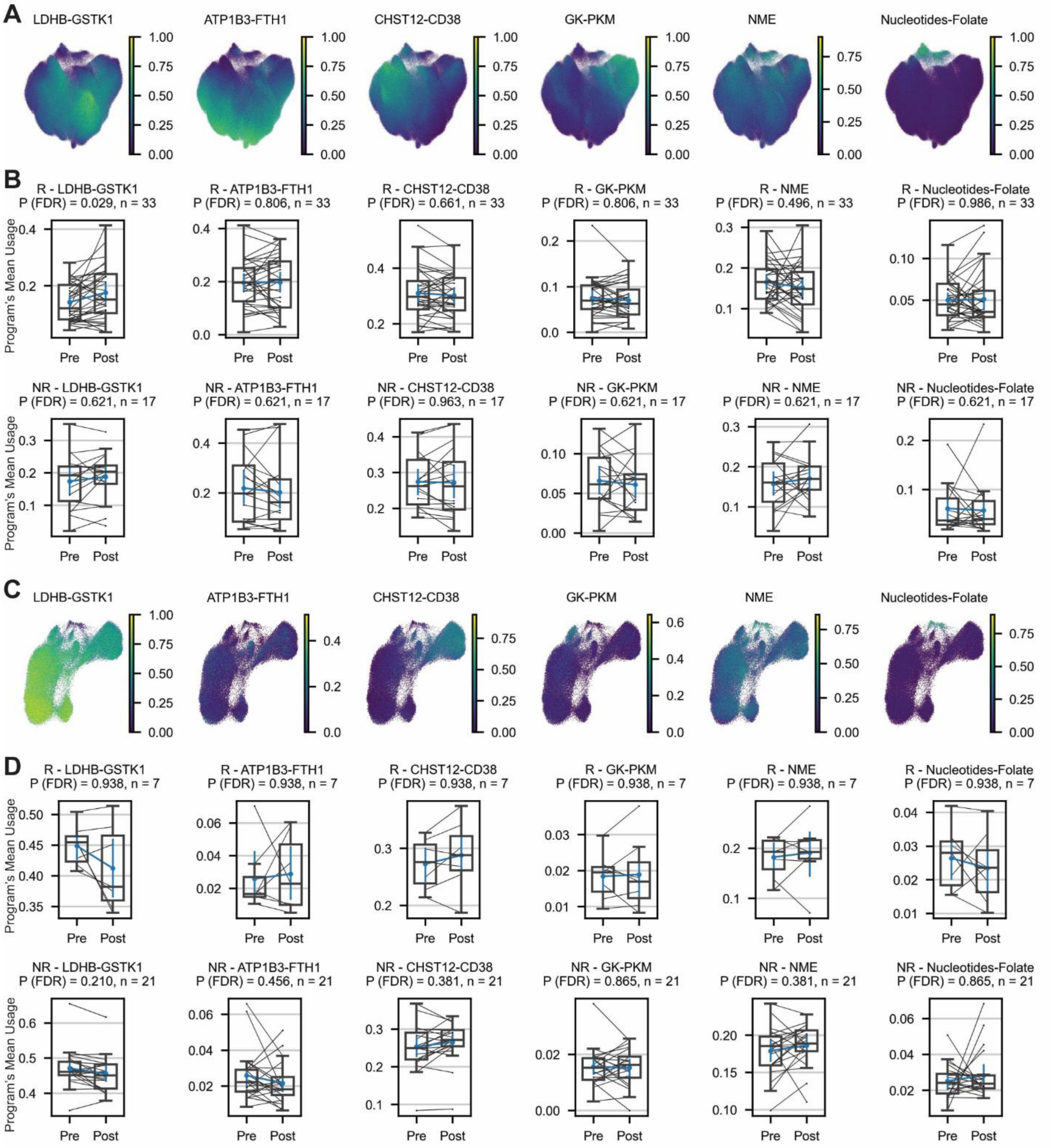

Figure S13. Changes following therapy of metabolic cNMF [S15] programs for the top 5 expanded persistent CD8$^+$ clones per patient in tumor and blood samples by clinical outcome, Related to Figure 3. A. Six metabolic programs obtained using cNMF and their usage across single cells from tumor samples. B. Changes in program activity following therapy for responders (top) and non-responders (bottom). C. Six metabolic programs obtained using cNMF and their usage across single cells from blood samples. D. Changes in program activity following therapy for responders (top) and non-responders (bottom). Abbreviations: R = Responders, NR = Non-responders.

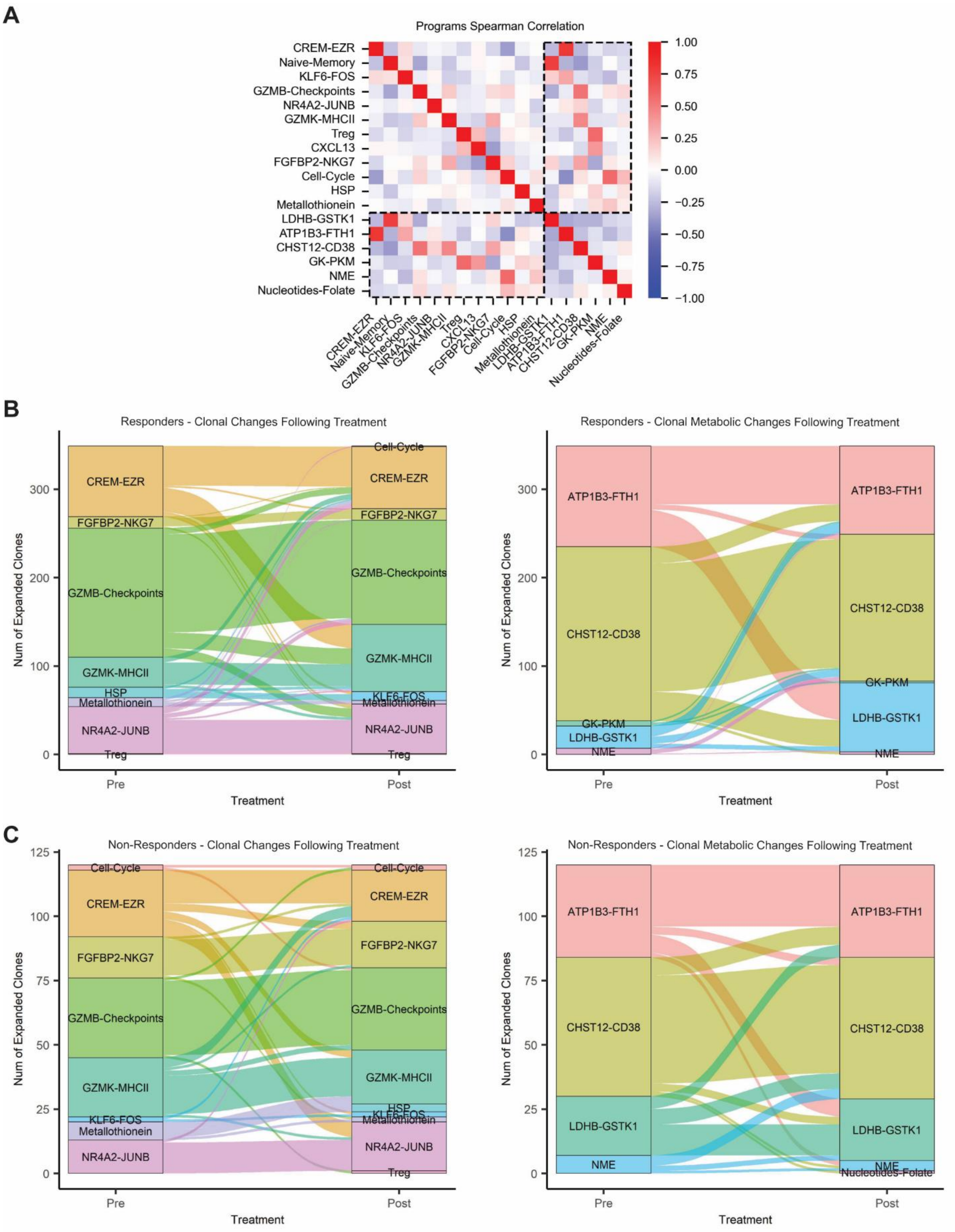

Figure S14. Changes following therapy of dominant cNMF [S15] programs for expanded persistent CD8⁺ clones in tumor samples, Related to Figure 3. A. Spearman correlation between twelve programs

and six metabolic programs across all single cells. B. Changes following therapy per clone for responders of cNMF programs (left) and metabolic cNMF programs (right). C. Changes following therapy per clone for non-responders of cNMF programs (left) and metabolic cNMF programs (right).

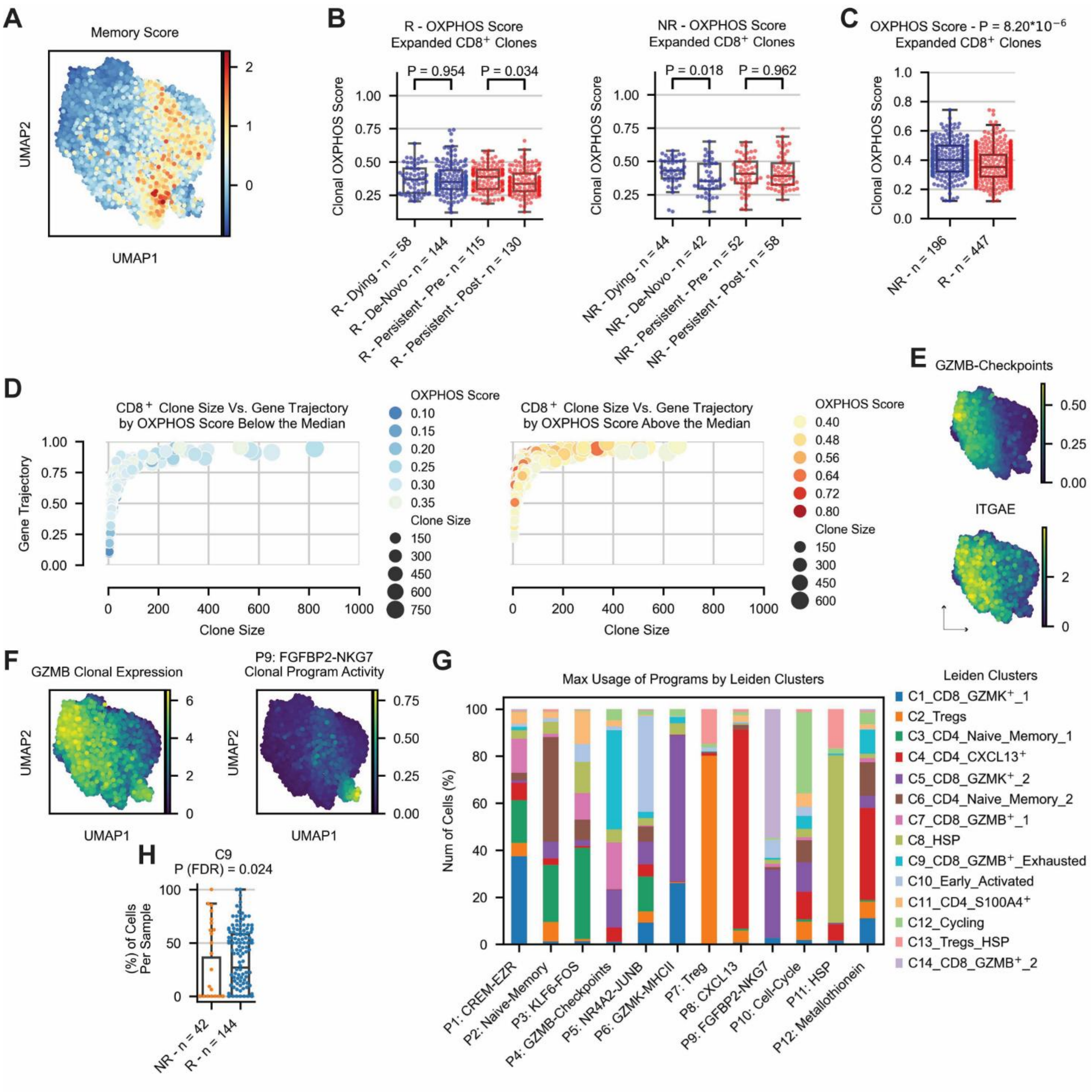

Figure S15. Pseudobulk analysis of expanded CD8⁺ clones, Related to Figure 4. A. Reflection of clonal memory score over the UMAP plot of 7,945 expanded CD8⁺ clones from tumor samples. B. Clonal oxidative phosphorylation score for the top 5 expanded CD8⁺ clones per sample in responders (left) and non-responders (right), separated by dying, de-novo and persistent clones. C. Clonal oxidative phosphorylation score for the top 5 expanded CD8⁺ clones per sample between responders and non-responders. D. Gene-trajectory value of each expanded CD8⁺ clone by its clone size, colored according to its clonal oxidative phosphorylation score. Clones with oxidative phosphorylation score below the median score are on the left, and those with score above the median are on the right. Clones with size < 1,000 cells used for visualization. E. UMAP plots of 7,945 expanded CD8⁺ clones from tumor samples showing the clonal usage of the GZMB-Checkpoints transcriptional program (top) and clonal expression of *ITGAE* (bottom). F. UMAP plots depicting the clonal expression of *GZMB* (left), as well as clonal activity of a *GZMB*-related transcriptional program (FGFBP2-NKG7, right). G. Composition of the max-usage within each transcriptional program by

14 Leiden clusters from tumor samples, demonstrating the activity of the GZMB-Checkpoints transcriptional program in C9, as well as in other *GZMB*-expressing clusters (C7 & C14). H. The abundance of single cells from C9 in de-novo clones by clinical outcome, emphasizing the importance of the contextual co-expression of *GZMB* in certain clusters or transcriptional programs. Abbreviations: OXPHOS = Oxidative phosphorylation, R = Responders, NR = Non-responders.

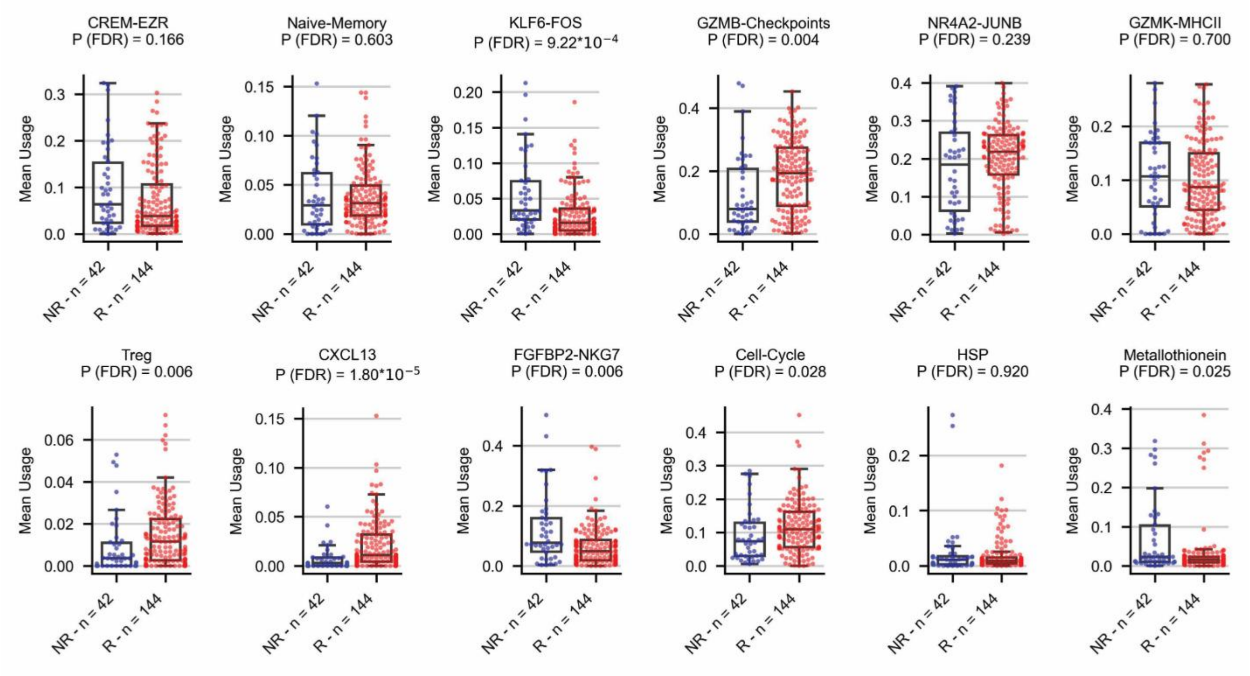

Figure S16. Changes of cNMF [S15] programs for the top expanded de-novo clones per tumor sample by clinical outcome, Related to Figure 4. Changes in program activity between de-novo clones in responders and non-responders. Abbreviations: R = Responders, NR = Non-responders.

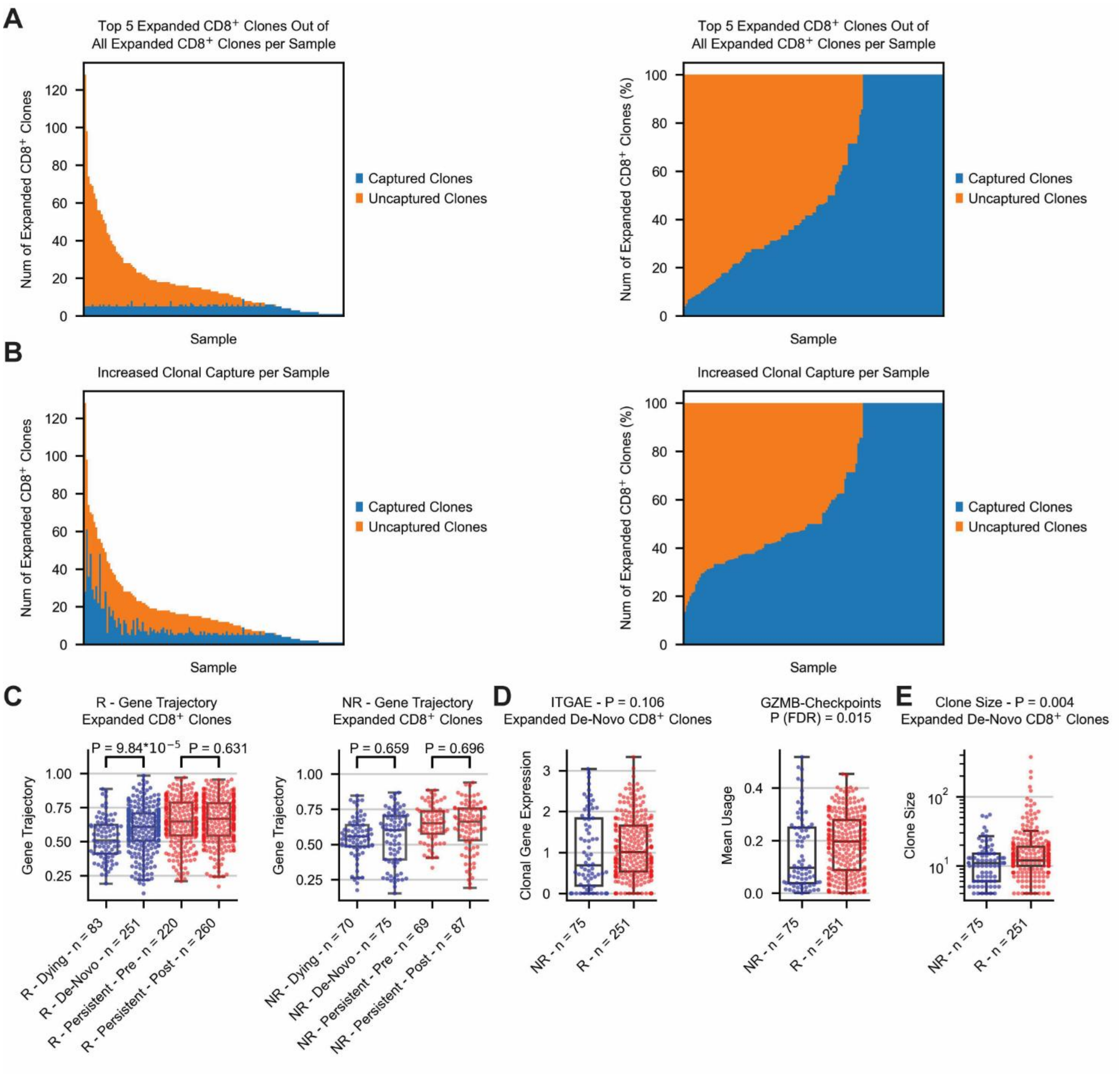

Figure S17. Pseudo-temporal analysis of expanded CD8+ clones following increased clonal capture per sample, Related to Figure 4. A. Original clonal capture per sample, including the absolute number of CD8+ clones that were considered (left), and the normalized amount per sample (right). B. Increased clonal capture per sample, including the absolute number of clones that were considered (left), and the normalized amount per sample (right). C. Gene-trajectory values for the increased capture of CD8+ clones per sample in responders (left) and non-responders (right), separated by dying, de-novo and persistent clones. D. Difference in *ITGAE* expression (left) and the activity of the GZMB-Checkpoints program (right) per clone, for the top expanded de-novo CD8+ clones in responders and non-responders. E. Difference in clone size for the top expanded de-novo CD8+ clones per sample between responders and non-responders. Abbreviations: R = Responders, NR = Non-responders.

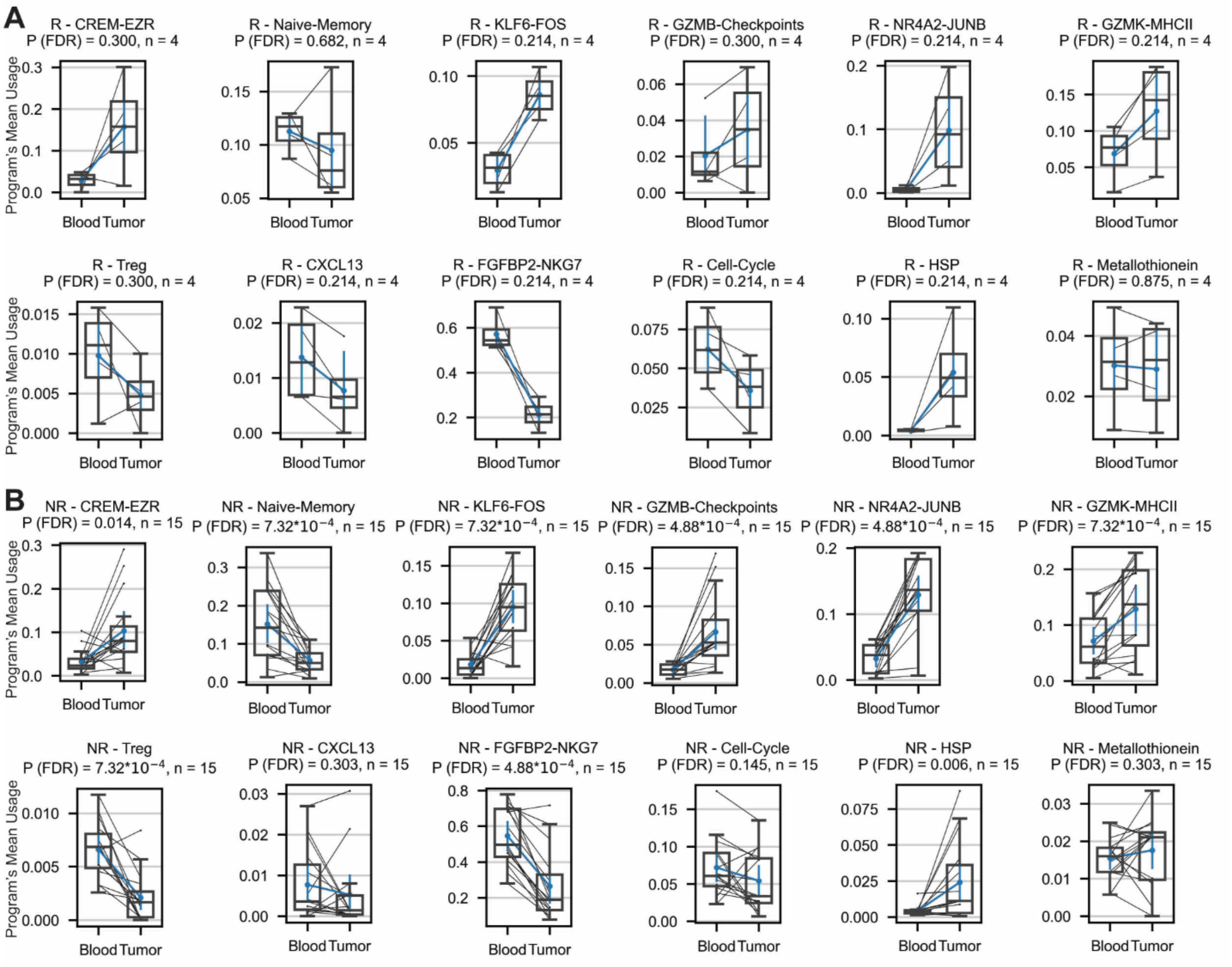

Figure S18. Changes of cNMF [S15] programs for the top 5 expanded CD8$^+$ clones per patient, of clones shared between tumor and blood samples, separated by clinical outcome, Related to Figure 5. A. Changes in program activity between tumor and matched blood samples for responders (n = 4 patients). B. Changes in program activity between tumor and matched blood samples for non-responders (n = 15 patients). Abbreviations: R = Responders, NR = Non-responders.

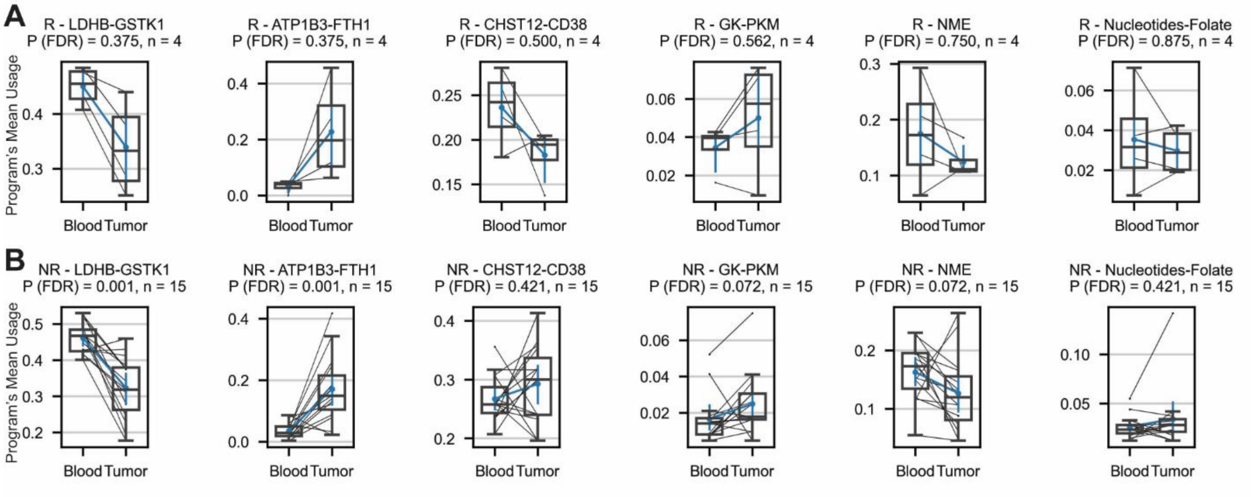

Figure S19. Changes of metabolic cNMF [S15] programs for the top 5 expanded CD8^{+} clones per patient, of clones shared between tumor and blood samples, separated by clinical outcome, Related to Figure 5. A. Changes in program activity between tumor and matched blood samples for responders (n = 4 patients). B. Changes in program activity between tumor and matched blood samples for non-responders (n = 15 patients). Abbreviations: R = Responders, NR = Non-responders.

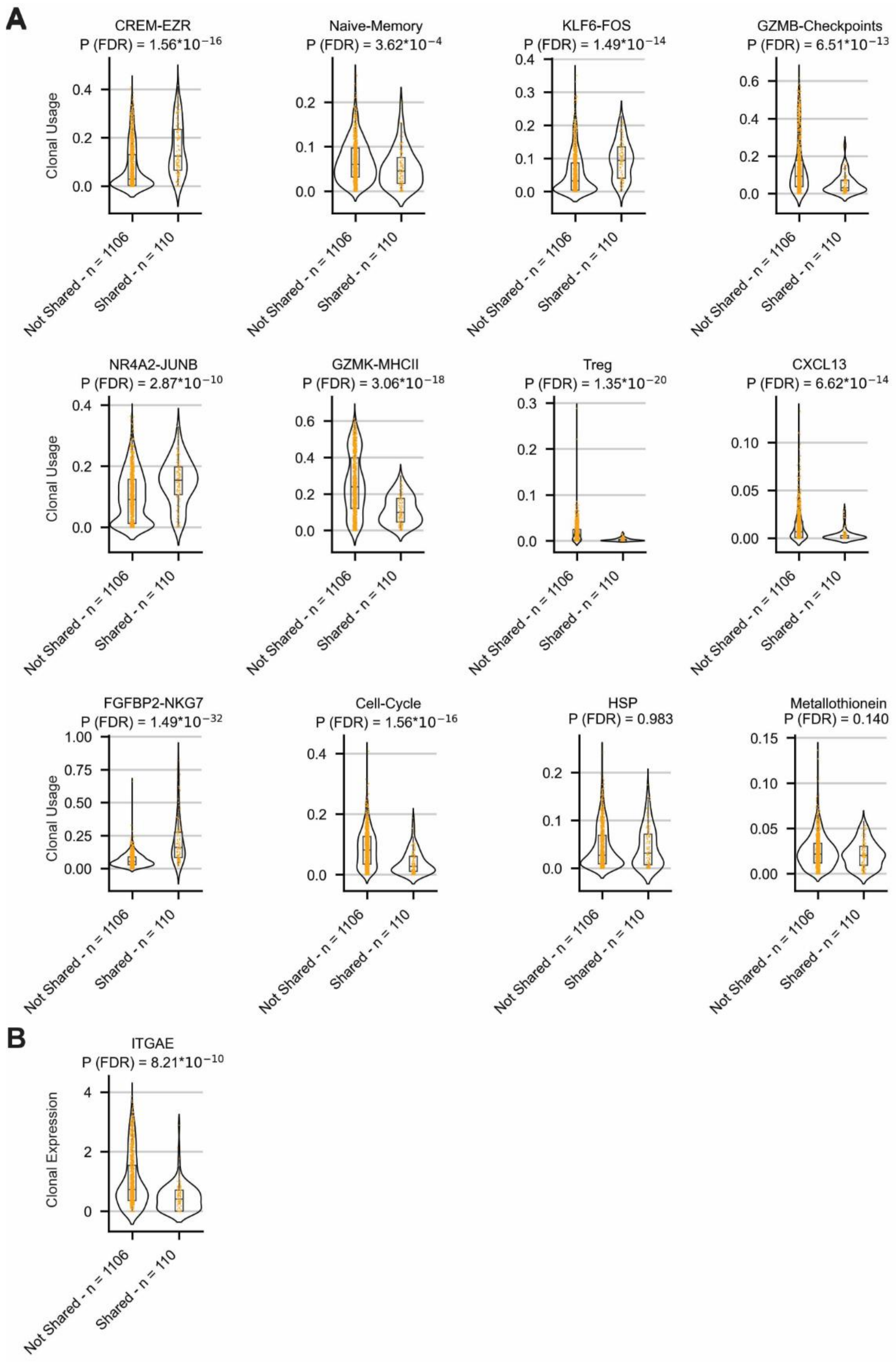

Figure S20. Clonal usage of cNMF [S15] programs in shared and non-shared clones, Related to Figure 5. A. Usage of cNMF programs per clone, for shared and non-shared clones. B. Clonal expression of *ITGAE* in shared and non-shared clones.

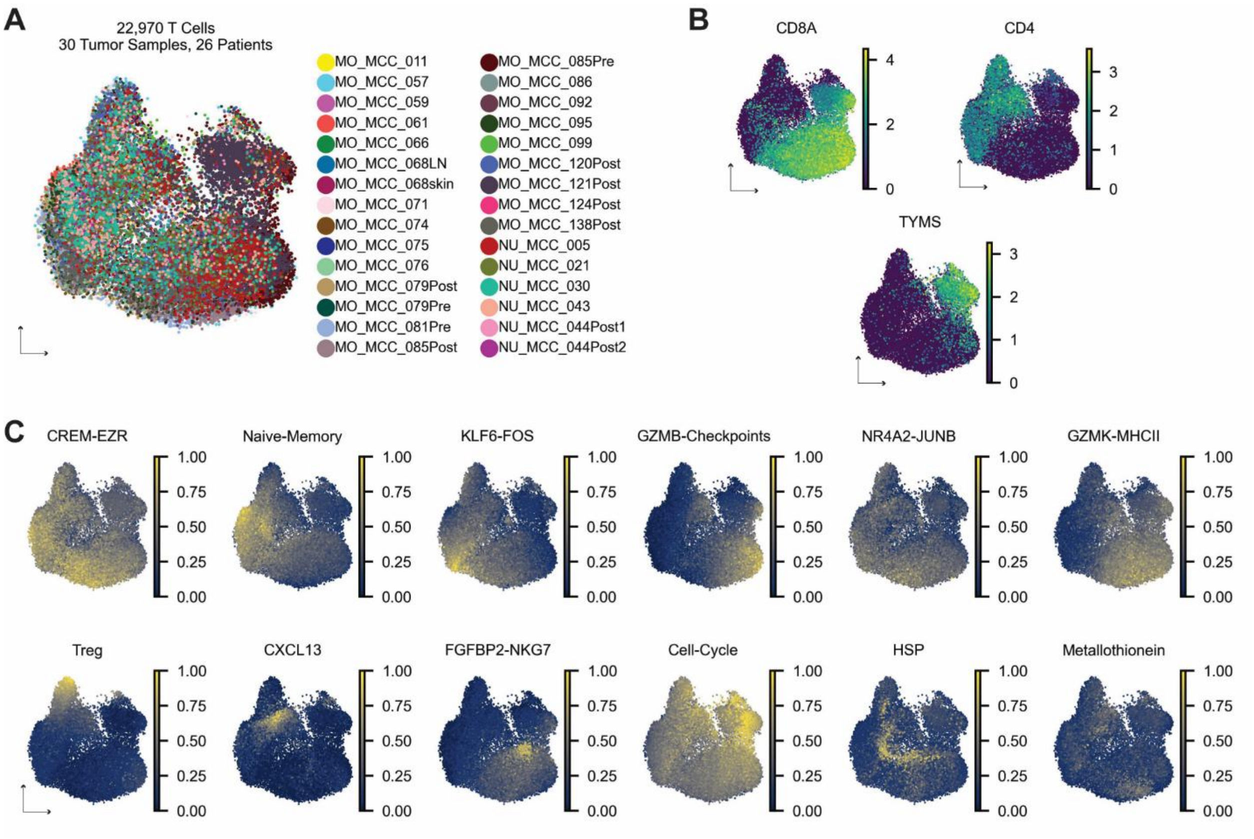

Figure S21. Validation scRNA/TCRseq dataset of ICI-treated MCC patients [S16], Related to Figure 5. A. UMAP plot showing 22,970 T cells passing our quality control, colored by attribution to MCC samples. B. UMAP plots colored by the expression of *CD8A*, *CD4* and *TYMS*. C. UMAP plots showing the projection of cNMF programs on the validation dataset using NMFproj [S17].

Supplemental references:

S1. van Dijk, D., Sharma, R., Nainys, J., Yim, K., Kathail, P., Carr, A.J., Burdziak, C., Moon, K.R., Chaffer, C.L., Pattabiraman, D., et al. (2018). Recovering Gene Interactions from Single-Cell Data Using Data Diffusion. Cell *174*, 716-729.e27. https://doi.org/10.1016/j.cell.2018.05.061.

S2. Bagaev, D.V., Vroomans, R.M.A., Samir, J., Stervbo, U., Rius, C., Dolton, G., Greenshields-Watson, A., Attaf, M., Egorov, E.S., Zvyagin, I.V., et al. (2020). VDJdb in 2019: database extension, new analysis infrastructure and a T-cell receptor motif compendium. Nucleic Acids Research *48*, D1057–D1062. https://doi.org/10.1093/nar/gkz874.

S3. Bassez, A., Vos, H., Van Dyck, L., Floris, G., Arijs, I., Desmedt, C., Boeckx, B., Vanden Bempt, M., Nevelsteen, I., Lambein, K., et al. (2021). A single-cell map of intratumoral changes during anti-PD1 treatment of patients with breast cancer. Nature Medicine *27*, 820–832. https://doi.org/10.1038/s41591-021-01323-8.

S4. Yost, K.E., Satpathy, A.T., Wells, D.K., Qi, Y., Wang, C., Kageyama, R., McNamara, K.L., Granja, J.M., Sarin, K.Y., Brown, R.A., et al. (2019). Clonal replacement of tumor-specific T cells following PD-1 blockade. Nat Med *25*, 1251–1259. https://doi.org/10.1038/s41591-019-0522-3.

S5. Liu, B., Hu, X., Feng, K., Gao, R., Xue, Z., Zhang, S., Zhang, Y., Corse, E., Hu, Y., Han, W., et al. (2022). Temporal single-cell tracing reveals clonal revival and expansion of precursor exhausted T cells during anti-PD-1 therapy in lung cancer. Nat Cancer *3*, 108–121. https://doi.org/10.1038/s43018-021-00292-8.

S6. Au, L., Hatipoglu, E., Robert de Massy, M., Litchfield, K., Beattie, G., Rowan, A., Schnidrig, D., Thompson, R., Byrne, F., Horswell, S., et al. (2021). Determinants of anti-PD-1 response and resistance in clear cell renal cell carcinoma. Cancer Cell *39*, 1497-1518.e11. https://doi.org/10.1016/j.ccell.2021.10.001.

S7. Bagley, S.J., Binder, Z.A., Lamrani, L., Marinari, E., Desai, A.S., Nasrallah, M.P., Maloney, E., Brem, S., Lustig, R.A., Kurtz, G., et al. (2024). Repeated peripheral infusions of anti-EGFRvIII CAR T cells in combination with pembrolizumab show no efficacy in glioblastoma: a phase 1 trial. Nat Cancer *5*, 517–531. https://doi.org/10.1038/s43018-023-00709-6.

S8. Pai, J.A., Hellmann, M.D., Sauter, J.L., Mattar, M., Rizvi, H., Woo, H.J., Shah, N., Nguyen, E.M., Uddin, F.Z., Quintanal-Villalonga, A., et al. (2023). Lineage tracing reveals clonal progenitors and long-term persistence of tumor-specific T cells during immune checkpoint blockade. Cancer Cell *41*, 776-790.e7. https://doi.org/10.1016/j.ccell.2023.03.009.

S9. Ganesan, A.-P., Clarke, J., Wood, O., Garrido-Martin, E.M., Chee, S.J., Mellows, T., Samaniego-Castruita, D., Singh, D., Seumois, G., Alzetani, A., et al. (2017). Tissue-resident memory features are linked to the magnitude of cytotoxic T cell responses in human lung cancer. Nat Immunol *18*, 940–950. https://doi.org/10.1038/ni.3775.

S10. Shiao, S.L., Gouin, K.H., Ing, N., Ho, A., Basho, R., Shah, A., Mebane, R.H., Zitser, D., Martinez, A., Mevises, N.-Y., et al. (2024). Single-cell and spatial profiling identify three response trajectories to pembrolizumab and radiation therapy in triple negative breast cancer. Cancer Cell *42*, 70-84.e8. https://doi.org/10.1016/j.ccell.2023.12.012.

S11. Luoma, A.M., Suo, S., Wang, Y., Gunasti, L., Porter, C.B.M., Nabilsi, N., Tadros, J., Ferretti, A.P., Liao, S., Gurer, C., et al. (2022). Tissue-resident memory and circulating T cells are early responders to pre-surgical cancer immunotherapy. Cell *185*, 2918-2935.e29. https://doi.org/10.1016/j.cell.2022.06.018.

S12. Zhang, Y., Chen, H., Mo, H., Hu, X., Gao, R., Zhao, Y., Liu, B., Niu, L., Sun, X., Yu, X., et al. (2021). Single-cell analyses reveal key immune cell subsets associated with response to PD-L1 blockade in triple-negative breast cancer. Cancer Cell *39*, 1578-1593.e8. https://doi.org/10.1016/j.ccell.2021.09.010.

S13. Barras, D., Ghisoni, E., Chiffelle, J., Orcurto, A., Dagher, J., Fahr, N., Benedetti, F., Crespo, I., Grimm, A.J., Morotti, M., et al. (2024). Response to tumor-infiltrating lymphocyte adoptive therapy is associated with preexisting CD8+ T-myeloid cell networks in melanoma. Science Immunology *9*, eadg7995. https://doi.org/10.1126/sciimmunol.adg7995.

S14. Krishna, C., DiNatale, R.G., Kuo, F., Srivastava, R.M., Vuong, L., Chowell, D., Gupta, S., Vanderbilt, C., Purohit, T.A., Liu, M., et al. (2021). Single-cell sequencing links multiregional immune landscapes and tissue-resident T cells in ccRCC to tumor topology and therapy efficacy. Cancer Cell *39*, 662-677.e6. https://doi.org/10.1016/j.ccell.2021.03.007.

S15. Kotliar, D., Veres, A., Nagy, M.A., Tabrizi, S., Hodis, E., Melton, D.A., and Sabeti, P.C. (2019). Identifying gene expression programs of cell-type identity and cellular activity with single-cell RNA-Seq. eLife *8*, 1–26. https://doi.org/10.7554/eLife.43803.

S16. Reinstein, Z.Z., Zhang, Y., Ospina, O.E., Nichols, M.D., Chu, V.A., Pulido, A. de Mingo., Prieto, K., Nguyen, J.V., Yin, R., Moran Segura, C., et al. (2024). Pre-existing skin-resident CD8 and γδ T cell circuits mediate immune response in Merkel cell carcinoma and predict immunotherapy efficacy. Cancer Discovery. https://doi.org/10.1158/2159-8290.CD-23-0798.

S17. Yasumizu, Y., Takeuchi, D., Morimoto, R., Takeshima, Y., Okuno, T., Kinoshita, M., Morita, T., Kato, Y., Wang, M., Motooka, D., et al. (2024). Single-cell transcriptome landscape of circulating CD4+ T cell populations in autoimmune diseases. Cell Genomics *4*, 100473. https://doi.org/10.1016/j.xgen.2023.100473.
